# Photoactivatable metal complexes: from theory to applications in biotechnology and medicine

**DOI:** 10.1098/rsta.2012.0519

**Published:** 2013-07-28

**Authors:** Nichola A. Smith, Peter J. Sadler

**Affiliations:** Department of Chemistry, University of Warwick, Coventry CV4 7AL, UK

**Keywords:** metal complexes, excited states, photochemistry, phototherapy, imaging, theory and computation

## Abstract

This short review highlights some of the exciting new experimental and theoretical developments in the field of photoactivatable metal complexes and their applications in biotechnology and medicine. The examples chosen are based on some of the presentations at the Royal Society Discussion Meeting in June 2012, many of which are featured in more detail in other articles in this issue. This is a young field. Even the photochemistry of well-known systems such as metal–carbonyl complexes is still being elucidated. Striking are the recent developments in theory and computation (e.g. time-dependent density functional theory) and in ultrafast-pulsed radiation techniques which allow photochemical reactions to be followed and their mechanisms to be revealed on picosecond/nanosecond time scales. Not only do some metal complexes (e.g. those of Ru and Ir) possess favourable emission properties which allow functional imaging of cells and tissues (e.g. DNA interactions), but metal complexes can also provide spatially controlled photorelease of bioactive small molecules (e.g. CO and NO)—a novel strategy for site-directed therapy. This extends to cancer therapy, where metal-based precursors offer the prospect of generating excited-state drugs with new mechanisms of action that complement and augment those of current organic photosensitizers.

## 1. Introduction

The absorption of ultraviolet and visible light by molecules can dramatically influence their reactivity. Excited-state molecules have different electronic distributions compared with ground states and therefore different geometries, bond angles and bond lengths. For example, in the excited states of transition metal complexes, the metal–ligand bond lengths can elongate (often selectively), not only making ligand release and exchange more facile, but also selective. The effects of electronic excitation on ligand exchange rates can be dramatic: substitution reactions on some relatively inert metal ions which might take hours in the ground state can occur within nanoseconds in excited states. Electronic excitation leading to the population of higher energy levels can be followed, not only by emission (fluorescence) from singlet states and decay back to the ground state, but also by the decay of long-lived states (phosphorescence) that are formed from singlet excited states via intersystem crossing (sometimes promoted by spin–orbit coupling). In turn, triplet states may have their own differences in geometry compared with singlet excited states and ground states.

**Figure 1.**
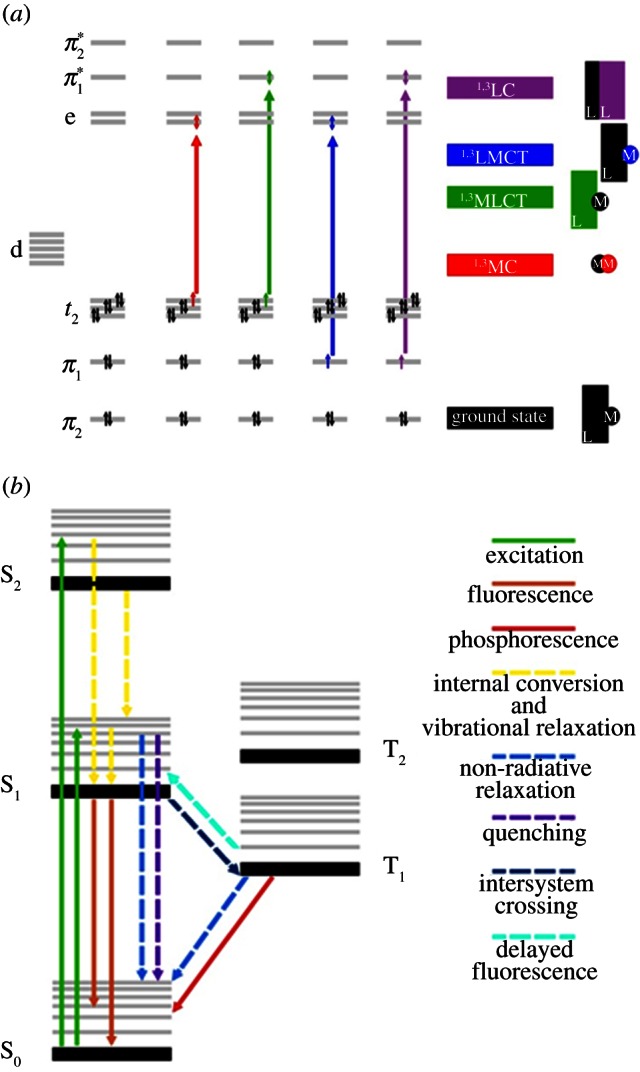
(*a*) Simplified orbital and excited-state diagram for a d^6^ metal complex with octahedral coordination (strong crystal field is assumed). Each black arrow (↑↓) represents an electron with its associated spin. Arrows represent the electron involved in each electronic transition. In the singlet state, electrons are spin down, whereas in the triplet state, they are spin up. (*b*) Jabłonski energy level diagram. Possible physical processes triggered by light excitation of a d^6^ metal complex are represented by dashed (radiationless) and solid (radiative) lines. Reproduced by permission of the Royal Society of Chemistry from Farrer *et al.* [1]. (Online version in colour.)

For transition metal complexes, a wide variety of electronic transitions are possible. These can be metal-centred (MC), ligand-centred (LC) or involve both the metal and the ligands: metal-to-ligand charge transfer, MLCT (for readily oxidized metal ions and ligands with low-lying acceptor orbitals), or ligand-to-metal charge transfer, LMCT (for readily reduced metal ions with strong donor ligands; figure 1). Charge-transfer transitions are usually intense and give rise to the possibility of complete transfer of an electron from metal to ligand or vice versa in the excited state and to irreversible photodecomposition (redox) reactions.

**Figure 2.**
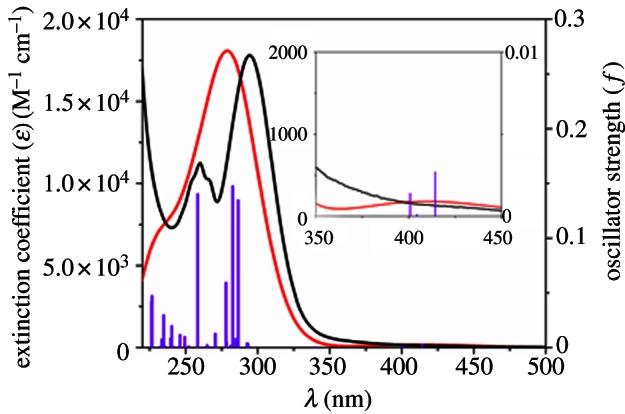
Calculated (grey) and experimental (black) absorption spectrum of the Pt^IV^ photoactivatable anti-cancer complex *trans,trans,trans*-[*Pt*(N_3_)_2_(OH)_2_(py)_2_] in H_2_O. The excited states are shown as vertical bars with heights equal to the extinction coefficients. According to TDDFT, the two absorption bands in the UV region can be assigned to dissociative ^1^LMCT (*N*_3_→Pt) and mixed ^1^LMCT/^1^*IL* (OH→Pt,N_3_, where IL=intra-ligand) transitions. The low intensity transitions in the blue region of the spectrum are dissociative with mixed ^1^LMCT/^1^IL character, involving N_3_ and OH ligands and Pt. Such transitions might account for the photoactivity induced by blue light. Adapted from Farrer *et al.* [2]. (Online version in colour.)

In general, the photochemistry of metal coordination complexes is poorly understood. Progress is currently gaining pace through the development, on the one hand, of theory, especially time-dependent density functional theory (TDDFT), and, on the other, of techniques for monitoring the population of excited electronic states and their accompanying structural changes, and for tracking photo-induced decomposition pathways. Pulsed radiation sources (e.g. lasers, synchrotron beams) are beginning to allow photochemical events to be followed on nano-, pico- and even femtosecond time scales. Moreover, the tunability of light sources now allows electronic transitions to be selectively activated. Density functional theory (DFT) calculations on transition metal complexes often show how a broad absorption band can conceal beneath it several electronic transitions. An example is shown in figure 2.

Fast lasers can deliver multiple pulses in rapid succession, allowing longer wavelength light to be used to activate higher energy transitions through the population of virtual electronic excited states (e.g. two- and three-photon absorption). This can have advantages where deeper penetration of the longer wavelength light is needed (e.g. into tissues for therapy) and where confinement of the photoactivation to *ca* femtolitre volumes is required.

Perhaps the widest appreciation of the importance of photochemistry in nature is in photosynthesis where plants and other organisms use pigments (especially chlorophyll, a metal complex containing magnesium) to capture energy from sunlight (*ca* 700 nm) to bring about the conversion of water to oxygen and carbon dioxide to sugars [3]. Another example of photochemistry of environmental importance concerns the bioavailability of iron in the oceans. This appears to be controlled on a large scale by the production of α-hydroxycarboxylic siderophores such as aquachelatin by marine bacteria which bind Fe^III^ tightly (and manifest a ligand-to-Fe^III^ charge-transfer band at *ca* 300 nm) and then undergo photochemical reduction to liberate Fe^II^ and CO_2_ [4,5]. This photochemistry of iron in the sea is reminiscent of that of the complex [Fe^*III*^(oxalate)_3_]^3−^, widely used as a chemical actinometer for determinations of quantum yields of photochemical reactions [6]. Probably the best known example of essential photochemistry for humans is the synthesis of vitamin D, required to produce hormones which regulate the concentrations of calcium and phosphate for healthy bones and to avoid osteomalacia (rickets). Vitamin D is synthesized photochemically in the skin from the steroid 7-dehydrocholesterol by the action of sunlight (UV) [7].

Selected examples will now be used to illustrate progress in the rapidly advancing field of inorganic and especially metal-based photochemistry. A particular emphasis is placed on current work which has potential applications in biotechnology and medicine.

## 2. Theory, computation and ultrafast detection

Current advances in the use of computational methods, especially DFT and TDDFT, are providing insights into the electronic transitions responsible for the absorption bands of transition metal complexes and their decomposition pathways. Even more powerful is the combination of theory and computation with fast detection methods which allow the decay of very short-lived excited states and their structures to be probed. For example, population of the antibonding MC orbitals in the ^3^MC state, in which the Ru–L bond is stretched, appears to be responsible for amine loss from [Ru(bpy)_2_(*L*)_2_]^2+^ (where bpy=2,2′-bipyridine; *L*=amine, e.g. pyridine) complexes. Visible excitation of the metal complex into its ^1^MLCT state in the visible region is followed by intersystem crossing into the ^3^MLCT state; subsequent mixing of the ^3^MLCT and ^3^MC states facilitates the population of the dissociative ^3^MC state [8]. Typically, DFT calculations can include the effects of solvation using, for example, the polarizable continuum model which can be implemented using the Gaussian program [9]. Fry & Mascharak [10] have used DFT and TDDFT calculations to reveal the key structural features required for the photolability of NO in metal nitrosyls derived from ligands containing carboxamido donors.

Metal carbonyl complexes have long been the subject of photochemical studies, but fast methods such as picosecond time-resolved infrared spectroscopy can reveal new features of such reactions. For example, the reaction pathways for [(η^6^-benzene)Mo(CO)_3_] depend on the excitation wavelength; use of 400 nm light populates a metal-to-arene charge-transfer excited state with liberation of CO on a 500 fs time scale, in contrast to the recovery of the initial complex with 266 nm excitation [11].

Transient species formed by 400 nm photoexcitation of aqueous [Ru(dppz)(tap)_2_]^2+^ (where dppz=*dipyrido*[3,2-*a*:2′,3′-*c*]phenazine; *tap*=1,4,5,8-tetraazaphenanthrene) when intercalated into [poly(dG-dC)]_2_ have been observed on a picosecond time scale by transient visible absorption spectroscopy, which allows the monitoring of metal complex intermediates, and transient infrared absorption spectroscopy that allows the direct study of DNA nucleobases. Excitation of the Ru^II^ complex when bound to [poly(dG-dC)]_2_ leads to the formation of the reduced complex [*Ru*^I^(dppz)(tap)_2_]^+^ and proton-coupled electron transfer processes involving G and C nucelobases [12].

The determination of the structures of transient species in light-induced processes presents a challenge for time-resolved techniques. For example, time-resolved wide angle X-ray scattering (TR-WAXS) has been used to study the photochemistry of *cis*-[Ru(*bpy*)_2_(py)_2_]^2+^. TR-WAXS can detect the release of a pyridine ligand and the coordination of a solvent molecule on a faster time scale than 800 ns of laser excitation. This ultrafast dissociation of pyridine (from a Ru^II^ complex which is highly inert in the ground state) appears to involve ^3^MLCT or ^3^MC states which decay on time scales of 100 ps to 5 ns, the former of which involves elongation and weakening of the Ru–N(py) bond [13].

We mention briefly now three recent advances in methods for delivery of light to samples.

## 3. Light delivery

The choice of the wavelength of light to activate complexes is not an easy decision. Firstly, the light source must be efficient in activating the complex. Often it is desirable to irradiate the complex where an intense MLCT band is present. However, this may not be practical for use in the clinic. Ultraviolet light is potentially harmful and so is not favoured. Red light penetrates deeper into tissue so is usually preferred. Two-photon activation using laser pulses occurs when two photons, usually at a wavelength of more than 600 nm, are sequentially absorbed to achieve excitation that a single photon at a shorter wavelength (approximately half) can achieve. Three-photon absorption is also achievable. This offers the possibility of activating complexes which absorb at a short wavelength using longer wavelengths of laser light which penetrate tissues deeply.

**Figure 3.**
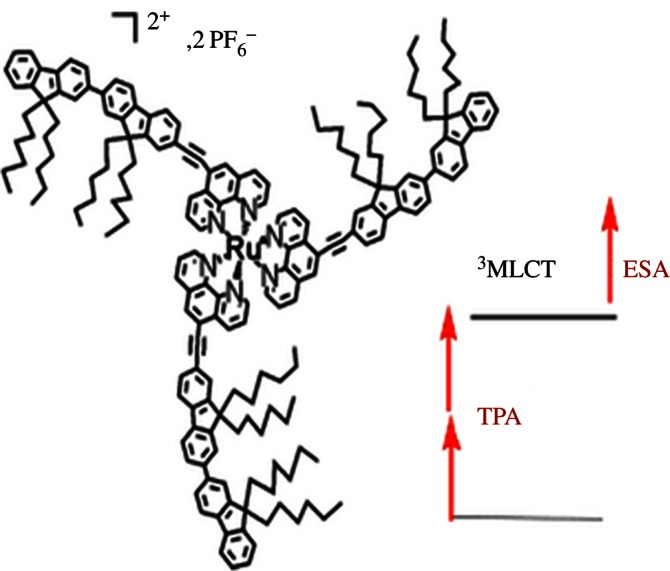
Two-photon absorption (TPA) in the singlet metal-to-ligand charge-transfer (^1^MLCT) band and subsequent excited-state absorption (ESA) allow highly conjugated ruthenium(II) systems, such as that shown, to be used for their optical power-limiting properties. Reproduced from Girardot *et al.* [15] with permission from John Wiley and Sons. (Online version in colour.)

The rules for designing metal complexes which can absorb two photons are not fully understood, but often large complexes of the type D–M–A which contain highly conjugated donor (D) and acceptor (A) ligands bound to the metal (M) are suitable. For example, Ru^II^ complexes containing highly conjugated fluorene-substituted 1,10-phenanthroline ligands exhibit two-photon activation between 600 and 700 nm, with a two-photon cross section of *ca* 530 GM, which is an order of magnitude higher than that for the ligand alone [14]. The two-photon absorption of Ru^II^ bifluorene-substituted 1,10-phenanthroline complexes is closely related to that of the ligand (figure 3) [15]. The introduction of extended conjugation into the amine ligands of square–planar Pt(II) complexes has allowed two-photon activation of ligand exchange using red and near-infrared (NIR) light. Interestingly, the optimum wavelength for two-photon activation of *cis*-[*PtCl*_2_(MOPEP)_2_], where MOPEP is the *π*-conjugated ligand 4-[2-(4-methoxyphenyl)ethynyl]pyridine, is shorter than twice the single-photon absorption wavelength [16].

**Figure 4.**
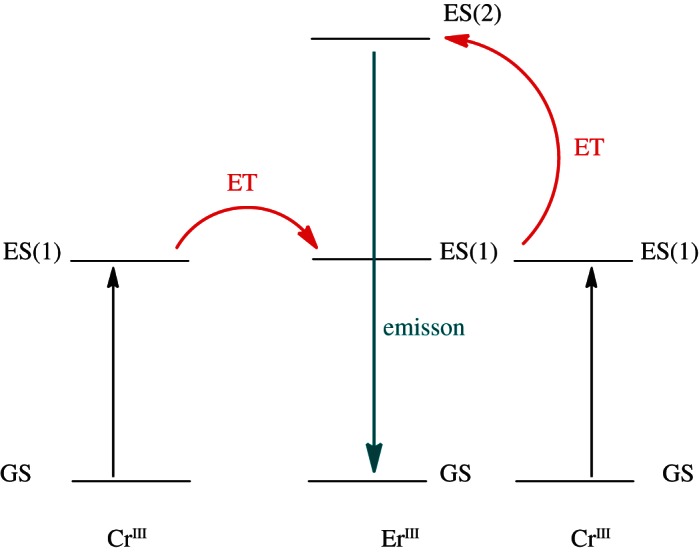
Illustration of upconversion of [CrErCr(*L*)_3_]^9+^ (L=a segmented polydentate ligand) to produce green light emission. Both chromium(III) centres are excited by low-energy irradiation from their ground state (GS) to their excited states (ES(1)). From here, the europium(III) centre undergoes two-photon upconversion via energy transfer (ET) from the chromium(III) centres to the europium(III) centre. As the excited state of europium(III) (ES(2)) decays, green light is emitted. Adapted from Aboshyan-Sorgho *et al.* [18]. (Online version in colour.)

Another promising method of producing shorter wavelength light from longer wavelength irradiation involves the use of upconverting nanoparticles, for example, made of YF_3_ doped with lanthanide ions (Yb^3+^ and Tm^3+^). Ford *et al.* have used lanthanide-doped upconversion nanoparticles to mediate nitric oxide (NO) release from Roussin's black salt anion [Fe_4_*S*_3_(NO)_7_]^−^, using NIR light from a simple diode laser operating at 980 nm [17]. In another example, the connection of two Cr^III^ sensitizers around a central Er^III^ acceptor in a self-assembled cation provides high local metal concentrations that favour efficient nonlinear energy transfer and upconversion luminescence (figure 4). Upon selective low-energy NIR irradiation of Cr^III^-centred transitions, the complex exhibits molecular two-photon green Er^III^-centred emission [18].

**Figure 5.**
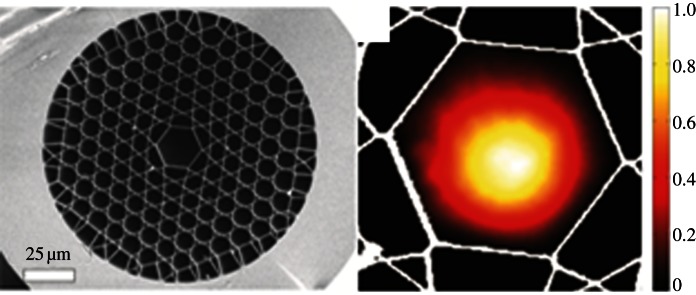
Scanning electron micrograph showing the cross section of a kagome hollow-core photonic crystal fibre. Adapted from Chen *et al.* [19] with permission from John Wiley and Sons. (Online version in colour.)

Hollow-core photonic crystal fibres (figure 5) allow photochemical reactions to be carried out efficiently on a nanolitre scale. Long optical path lengths are possible as a result of very low intrinsic waveguide loss. The light travels in a diffractionless single mode with a constant transverse intensity profile. For example, the photochemical conversion of vitamin B_12_ (cyanocobalamin, CNCbl) to hydroxocobalamin ([*H*_2_OCbl]^+^) in aqueous solution, a reaction with very low quantum yield, requires 10^4^ times less sample volume compared with conventional techniques and occurs 10^3^ times faster than in a conventional cuvette [19].

## 4. Photo-induced release of bioactive molecules

There is interest in the delivery of small molecules which can act as second messengers and transmit signals into cells. Examples include NO, carbon monoxide (CO) and hydrogen sulfide (H_2_S).

**Figure 6.**
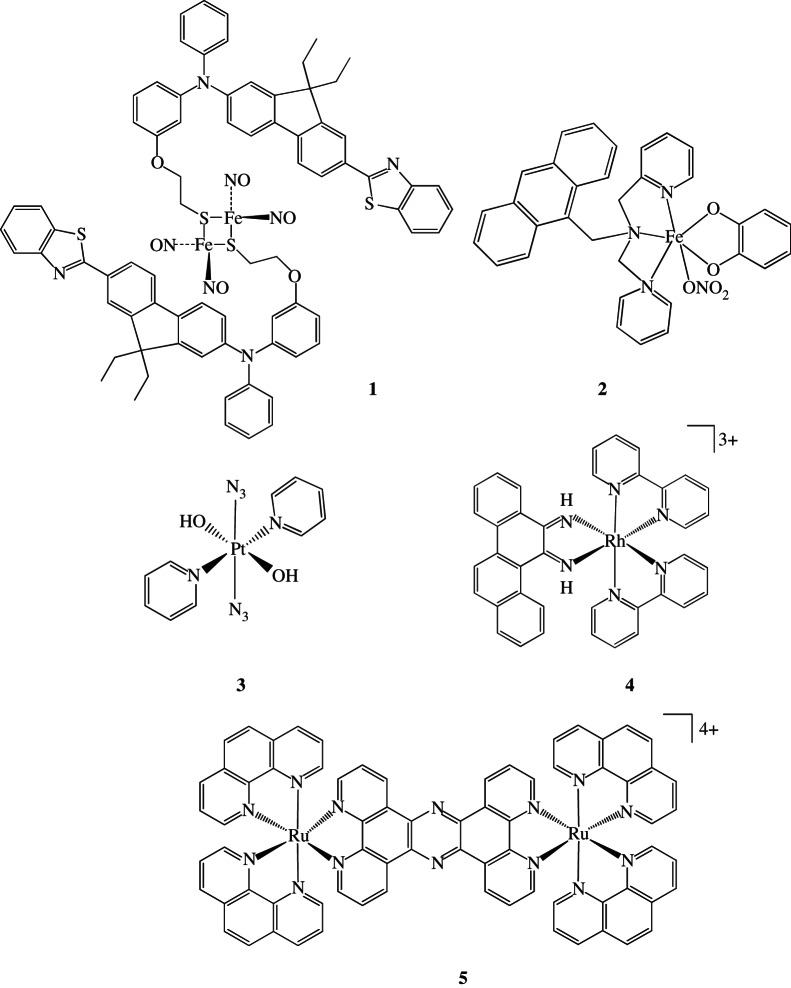
Various photoactivatable metal complexes.

NO is synthesized by the enzyme NO synthase in the endothelium of blood vessels and causes the surrounding smooth muscle to relax, resulting in vasodilation. It is a highly reactive gas but can be stabilized by binding to metal ions, and released from some complexes by photoactivation, for example, Roussin's red salt ester (RSE, [Fe_2_(μ-RS)_2_(*NO*)_4_]). Attachment of a chromophore to RSE can enhance the light harvesting ability of the complex and allow it to be activated by longer wavelength light. There is efficient energy transfer from the excited state of the chromophore, *N*-phenyl-*N*-(3-(2-ethoxy)phenyl)-7-(benzothiazol-2-yl)-9,9-diethylfluoren-2-ylamine, to the metal centre (complex **1** in figure 6) [20].

Ford *et al*. have shown that porphyrins which absorb strongly in the visible region can act as antennae to improve the photoactivity of metal complexes, such as RSE, [Fe_2_(μ-SEt)_2_(*NO*)_4_]. Upon photoactivation in the Q band of the porphyrin, NO is released at a higher rate compared with that for RSE alone. The porphyrin complex can be activated by a femtosecond pulsed NIR laser at 810 nm, allowing two-photon activation [21]. Da Silva and co-workers [22] have shown that lipid nanoparticles can be used to deliver [Ru(terpy)(bdqi)NO]^3+^ (terpy=*terpyridine*, bdqi=1,2-benzoquinone diimine) to its target via topical administration; this complex releases NO on irradiation with visible light for the possible treatment of skin cancer. Mascharak and co-workers [23] have shown that irradiation of the manganese nitrosyl complex [Mn(PaPy_3_)(NO)]^+^ (PaPy=*N*,*N*-bis(2-pyridylmethyl)amine-*N*-ethyl-2-pyridine-2-carboxamide), loaded into a biocompatible mobile material, with visible light can release NO and destroy drug-resistant *Acinetobacter baumannii* bacteria.

Photoactive metal complexes can be used to deliver CO which, like NO, is also a natural signalling molecule in the body [24]. For example, [Mn(tpa)(*CO*)_3_]^+^ (tpa=tris(2-pyridyl)amine) can release CO when irradiated by visible light. The sensitivity of the complex to light can be increased by extending the conjugation in the ligand, as in the Mn^*I*^ carbonyl complex [Mn(pqa)(*CO*)_3_]^+^ (pqa=(2-*pyridylmethyl*)(2-quinolylmethyl)amine) [25]. Functionalization of manganese complexes with peptides offers potential for the selective delivery of CO-releasing manganese complexes to cells [26]. Time-resolved IR spectroscopy is powerful for monitoring the release of CO from these complexes [27].

Besides the small signalling molecules NO and CO, photoactivation of metal complexes can be used to deliver other biologically active species such as the neurochemical amino acid γ-amino butyric acid (GABA). Octahedral Ru(II) complexes such as [Ru(bpy)_2_(*PPh*_3_)(GABA)]^2+^ can be activated by visible light and the released GABA can induce receptor-mediated responses in the brain [28]. The time constant for nicotine (Nic) release from [Ru(bpy)_2_(*Nic*)_2_]^2+^ is short, 17 ns [29]. Similarly, the complex [Ru(bpy)_2_(PMe_3_)(*GluH*_2_)]^2+^ releases glutamate within 50 ns on irradiation with visible light. Moreover, the latter complex is also activated by two-photon activation with 800 nm light [30].

For therapy, specific enzyme inhibitors can be generated by photoactivation. [Ru(bpy)(4*AP*)_4_]^2+^ (4AP=4-aminopyridine) preferentially liberates an equatorial 4AP molecule when irradiated at 488 nm [31]. [*Ru*(bpy)_2_(CPI)_2_]^2+^ releases (*S*)-2-acetamido-*N*-(cyanomethyl)-3-phenylpropanamide (CPI) on irradiation with white light which inhibits cathepsin, a cysteine protease, and represents a possible strategy for anti-cancer drug design [32]. On release of 5-cyanouracil (5CNU) from [Ru(*bpy*)_2_(5CNU)_2_]^2+^ on irradiation with visible light, the resulting aqua complex [Ru(*bpy*)_2_(H_2_O)_2_]^2+^ can bind to DNA. The complex can therefore have a dual mode of action through 5CNU release and DNA binding [33].

Photochemotherapy is currently used clinically and is normally termed photodynamic therapy (PDT). In PDT, the active species which kills cancer cells is singlet oxygen (^1^O_2_), produced as a result of photoactivation of a photosensitizer (e.g. a porphyrin) with light; the excited triplet state of the photosensitizer can then convert ground state ^3^*O*_2_ to ^1^O_2_. PDT is, therefore, effective when cells are rich in O_2_ although many tumours are relatively hypoxic. Red light is usually used for PDT because it penetrates into tissue more deeply than shorter wavelength light.

Some metal complexes can be activated by red or even NIR irradiation if an appropriate choice of ligands is made. For example, the vanadium curcumin complex [VO(cur)(dppz)Cl] (dppz=*dipyrido*[3,2-a:2′,3′-c]*phenazine*) is more active towards cancer cells than the clinical photosensitizer phorofrin when activated by visible light [34]. Similarly, the anthrocenyl ligand (L) in [Fe(L)(cat)NO_3_] (where *L*=9-[(2,2′-dipicolylamino)methyl]anthracene, *cat*=catecholate) acts as a photosensitizer and as a DNA intercalator (complex **2** in figure 6). The presence of the catecholate ligand provides IR absorption [35]. Photoactivated [Ru(tpy)(dppn)]^2+^ produces ^1^O_2_ very efficiently and leads to cross-links in the tumour suppressor protein p53, as well as producing protein–DNA cross-links [36].

Photoactive Pt^IV^ diazido complexes also offer potential dual mode activity; excited singlet and triplet states can release reactive or biologically active ligands and form Pt^II^ species which can bind to DNA [37]. For example, the diazido complex *trans,trans,trans*-[Pt(OH)_2_(*N*_3_)_2_(py)_2_] (py=pyridine) (**3** in figure 6) is stable in the dark even in the presence of the tripeptide glutathione (γ-l-glutamyl-l-cysteinylglycine), a reducing agent present in most cells at millimolar concentrations. This complex undergoes photoreduction when irradiated by UVA (320–400 nm), blue or green light and produces unusual cross-links on DNA [2,38]. These are probably interstrand *trans*-{Pt(*py*)_2_}^2+^ cross-links [39]. In addition, azidyl radicals can be trapped after photoactivation. Interestingly, these azidyl radicals can be quenched by l-tryptophan, a natural amino acid often given as a supplement, the administration of which might allow control of radical reactivity in cells [40]. The related diazido complex *trans,trans,trans*-[Pt(*OH*)_2_(N_3_)_2_(NH_3_)(py)] is active *in vivo* in an oesophageal cancer model [41]. These photoactive diazyl complexes are more active as *trans* isomers in contrast to current clinical platinum anti-cancer drugs which are all *cis*-diam(m)ine complexes. Two-photon absorption offers the possibility of activating platinum complexes that absorb at shorter wavelengths (e.g. in the UV region) using longer wavelengths (e.g. red light) that penetrate more deeply into tissues. For example, ligand substitution in square–planar Pt^II^ complexes containing conjugated amine ligands can be activated by two-photon absorption, but interestingly, the absorption maximum for one-photon absorption is displaced slightly from the two-photon wavelength [16].

## 5. Photo-triggered DNA binding and imaging

Targeting of metal complexes to tumour cells, or even to specific molecular targets in tumour cells, followed by photoactivation might be a very powerful strategy for the selective destruction of cancer cells. For example, the receptor-binding peptide Arg–Gly–Asp (RGD) has been incorporated as a substituent on a pyridine ligand in the ruthenium(II) arene complex [(η^6^-*p*-cym)Ru(bpm)(py-RGD)]^2+^. RGD receptors are overexpressed on cancer cells. Activation by visible light releases the peptide and gives the reactive aqua species, [(η^6^-*p*-cym)*Ru*(bpm)(H_2_O)]^2+^, which can bind to DNA. When irradiated in the presence of DNA, the complex can lose the *p*-cymene arene ligand and form a bi-functional adduct with two guanines [42].

Another useful property of Ru^II^ polypyridyl complexes is their luminescence. For example, the uptake and localization of [Ru(bpy)_2_(*dppz*)]^2+^ in cells can be tracked [43]. The complex [Ru(TAP)_2_PHEHAT]^2+^ interchalates into DNA in the dark, causing single strand breaks when irradiated with visible light [44]. Polyazaaromatic Ru^II^ complexes have potential as cellular diagnostics and photoreagents, especially for DNA attack and gene sequencing [45]. The dinuclear ruthenium(II) complex [(phen)_2_Ru(*tpphz*)Ru(phen)_2_]^4+^ (complex **5** in figure 6) is a DNA stain which can be imaged by techniques such as luminescence microscopy and transmission electron microscopy. The complex accumulates in the nucleus and stains heterochromatin within individual chromosomes [46].

The complex [Rh(bpy)_2_(chrysi)]^3+^ (complex **4** in figure 6) targets single-base mismatches in DNA by non-covalent binding and, on irradiation with UV/visible light, strand scission occurs. This complex can therefore identify mismatched sites. In cancer cells, there is often a deficiency of mismatch repair and this technique might be useful as a diagnostic tool for detecting cancer cells [47,48]. Also promising are N-heterocylic carbene (NHC) cyclometallated platinum(II) complexes, [(C^∧^N^∧^N)Pt(NHC)]^+^ (where C^∧^N^∧^N is a chelated C- and N-bound ligand), which are highly luminescent so their localization can be followed by emission microscopy. The complexes do not interact with DNA but localize in cytoplasmic structures [49].

Finally, we mention luminescent lanthanide probes and cyclometallated Ir^III^ complexes [50]. Luminescent cyclometallated iridium(III) polypyridine complexes containing di-2-picolylamine [Ir(N^∧^C)_2_(phen-DPA)](PF_6_) (phen-*DPA*=5-(di-2-picolylamino)-1,10-phenanthroline; HN^∧^C= 2-phenylpyridine) on photoexcitation can give rise to intense and long-lived luminescence. Lee *et al.* have assigned the emission to a triplet metal-to-ligand charge-transfer ^3^MLCT d*π*(Ir)→π* (N^∧^N) or to a triplet intraligand ^3^IL *π*→π* (N^∧^C) excited state, with substantial mixing of triplet amine-to-ligand charge-transfer ^3^NLCT *n*→π* (N^∧^N) character [51]. Luminescent cyclometallated iridium(III) polypyridine indole complexes, [Ir(N–C)_2_(N–N)](PF_6_), N–N=4-((2-(indol-3-yl)ethyl)aminocarbonyl)-4'-methyl-2,2'-bipyridine (bpy–ind), have an intense and long-lived luminescence (*λ*_em_=540–616 nm, *τ*=0.13–5.15 μs). In addition, the IC_50_ (dose which kills 50% of the cells) values of the complexes towards human cervix epithelioid carcinoma (HeLa) cells range from 1.1 to 6.3 μM, significantly more potent than cisplatin (30.7 μM) under the same experimental conditions. The cellular uptake of the complexes has been investigated by flow cytometry and laser-scanning confocal microscopy. The microscopy images indicated that the bpy–ind complex localizes in the perinuclear region [52].

## 6. Conclusions

Our brief review has highlighted some of the interesting new developments in the field of inorganic metal photochemistry many of which are discussed further in the following articles in this Discussion Meeting Issue. In particular, the photochemistry of metal complexes can make new contributions to biological imaging and to the design of photoactivated chemotherapeutic agents. Recent developments in theory and computation (e.g. TDDFT) and in ultrafast-pulsed radiation techniques now allow photochemical reactions to be followed on picosecond/nanosecond time scales, and the pathways for photodecomposition to be investigated and associated with specific electronically excited states. The choice of the metal ion, its oxidation state, the numbers and types of coordinated ligands and coordination geometry are all important to the design of photoactive complexes. Photochemical events in general involve not only the metal ion but also the ligands too. Some metal complexes possess favourable emission properties which allow functional imaging of cells and tissues (e.g. DNA interactions); they can also provide spatially controlled photorelease of bioactive small molecules (e.g. CO, NO, H_2_S, neurotransmitters and other signalling molecules) which can be exploited in site-directed therapy. For example, photoactivated metal-based anti-cancer pro-drugs offer the prospect of generating excited-state drugs with new mechanisms of action that can complement current organic photosensitizers.
